# Transcriptomic Analysis of Tendon Healing Using an Extracellular Matrix-Coated, Polyurethane Scaffold

**DOI:** 10.3390/bioengineering13060652

**Published:** 2026-05-31

**Authors:** Ying Rao, Marianne Lauwers, Shuting Huang, Yuyue Zhang, Dai Fei Elmer Ker, Rocky S. Tuan, Dan Michelle Wang

**Affiliations:** 1 School of Biomedical Sciences, Faculty of Medicine, The Chinese University of Hong Kong, Hong Kong SAR, China; yingrao@link.cuhk.edu.hk (Y.R.);; 2 Institute for Tissue Engineering and Regenerative Medicine, The Chinese University of Hong Kong, Hong Kong SAR, China; 3 Center for Neuromusculoskeletal Restorative Medicine, Hong Kong Science and Technology Park, Hong Kong SAR, China; marilauwers@cnrm.com.hk (M.L.);; 4 Department of Biomedical Engineering, The Hong Kong Polytechnic University, Hong Kong SAR, China; 5 Department of Orthopaedics and Traumatology, Faculty of Medicine, The Chinese University of Hong Kong, Hong Kong SAR, China

**Keywords:** large rotator cuff tendon defect, tendon regeneration, extracellular matrix, transcriptomic analysis, polyurethane scaffold

## Abstract

Large rotator cuff tendon injuries pose a dual clinical challenge: poor inherent healing capacity and high mechanical demands. To address this, we have developed a bifunctional scaffold that combines a slow-degrading, mechanically robust polyurethane core coated with tendon-derived extracellular matrix (ECM) extract to provide both structural support and regenerative cues. In a rabbit model of supraspinatus tendon injury, this ECM-polyurethane scaffold facilitated healing of critical-sized defects, resulting in aligned, tendon-like tissue with improved biomechanical properties. This study further explored the tendon healing mechanisms of the ECM-polyurethane scaffold in a rabbit model of large supraspinatus tendon injury using transcriptomic and qPCR analyses. At one month post-surgery, while both ECM-coated and uncoated polyurethane scaffolds initially provoked similar inflammatory responses when compared to healthy tendon, their healing pathways diverged significantly. The control polyurethane scaffold activated pathways associated with adipose tissue development, a non-functional outcome, whereas the ECM-coated scaffold actively directed healing toward tendon regeneration. These results demonstrate that the ECM coating is the critical factor driving divergent healing responses in polyurethane scaffolds, even though their underlying biomechanical properties are similar. This underscores the importance of combining biomechanical reinforcement with biologically active regenerative signals for effective regeneration of tendon and other load-bearing tissues.

## 1. Introduction

Tendons are dense fibrous connective tissue bands with high tensile strength that play a crucial role in facilitating force transmission during musculoskeletal movement [1,2]. Nevertheless, tendons are susceptible to injuries from acute (overload) and/or degenerative aging processes, and their poor innate regenerative capacity makes achieving full functional recovery difficult. Substantial tendon injuries, such as large rotator cuff tears (RCTs), present significant challenges for shoulder surgeons in clinical practice [1]. Despite remarkable improvements in surgical techniques over the past decades, the retear rate remains considerably high, ranging from 20 to 90% [1]. This high retear rate can be attributed to incomplete healing, characterized by disorganized extracellular matrix (ECM), the formation of fibrovascular scar tissue and inferior mechanical strength [3,4,5,6]. Consequently, achieving functional tendon repair requires biological and biomechanical augmentation to regenerate or replicate the native tendon-like tissue structure [2,7].

To address this challenge, our laboratory previously developed a hybrid ECM-polyurethane scaffold, which aimed to simultaneously achieve robust tendon regeneration and adequate mechanical support [8]. Specifically, we employed a urea-based extraction method to prepare a soluble, DNA-free, ECM fraction from bovine tendons that showed robust tenogenic bioactivity in vitro and in vivo [9,10]. To provide sufficient mechanical support for sustaining long-term shoulder movement, we developed an ultraviolet (UV)-crosslinkable polyurethane elastomer (QHM), which demonstrates biomechanical properties similar to the human supraspinatus tendon (SSPT), excellent suture retention, slow degradation, low cytotoxicity, and good biocompatibility [8,11]. These two aspects are integrated in the ECM-polyurethane scaffold through covalent crosslinking of an ECM-rich gelatin methacrylate (GelMA) hydrogel and the mechanically robust QHM. Our results demonstrate that the ECM-polyurethane scaffold significantly improved tendon regeneration and functional recovery of large tendon defects in rabbits. Notably, we observed a wavy configuration of tendon-like ECM structures, which contrasts sharply with the disorganized scar tissue formation observed in the control-polyurethane (ctrl-polyurethane) scaffold group [8].

Despite these encouraging results, the underlying molecular mechanisms by which the ECM-polyurethane scaffold promoted tendon regeneration remain poorly defined. This is particularly true regarding the differences in healing observed between our polyurethane scaffold with or without ECM coating [12,13,14]. A deeper mechanistic understanding is necessary to explain how scaffold design influences cell fate decisions and tissue regeneration, which in turn can guide the rational design and improvement of biomaterial therapies. Transcriptomic analysis provides valuable insights into these mechanisms, as mapping regulatory networks can identify actionable pathways for therapeutic targeting, support biomarker discovery, and inform individualized treatment strategies [12,13,14].

**Figure 1:**
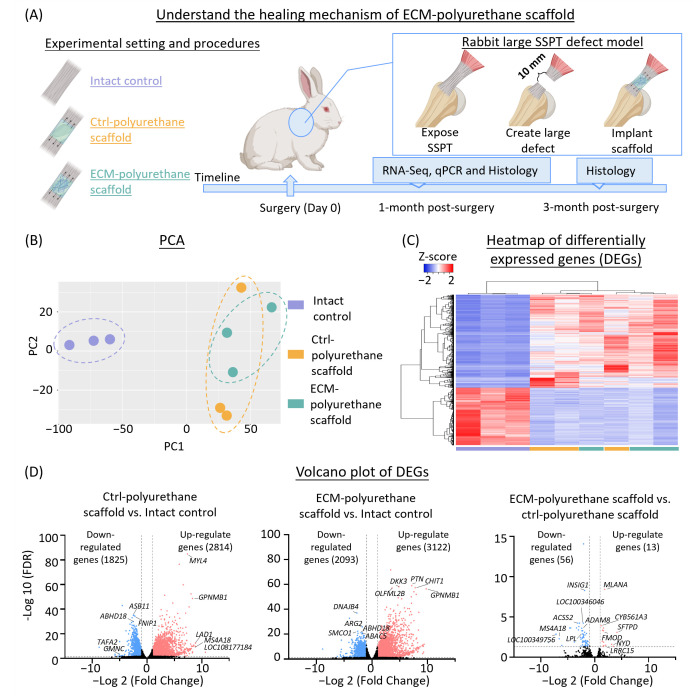
Study overview and global transcriptomic characterization of the intact control and ctrl- and ECM-polyurethane scaffold groups. (**A**) This study aims to elucidate the healing mechanisms of ECM-polyurethane scaffolds for repairing large tendon defects. A large tendon defect model was created on the supraspinatus tendon (SSPT) of New Zealand White rabbits. Briefly, a full-thickness defect (10 mm in length) was created, and different scaffolds (ctrl- and ECM-polyurethane scaffolds) were implanted at the defect sites. The contralateral shoulder was used as an intact control. One month post-surgery, RNA was isolated from the SSPT for RNA-Seq and qPCR analysis. Moreover, histology analysis was performed at 1 and 3 months post-surgery time point. (**B**) PCA revealing notable variance in transcriptional profile among the three groups with the primary source of variation associated with scaffold implantation. (**C**) Heatmap illustrating transcriptomic results for DEGs among the intact control and ctrl- and ECM-polyurethane scaffold groups. (**D**) Volcano plots depicting upregulated and downregulated DEGs (dashed lines: log_2_ fold change > |1| and false discovery rate (FDR) < 0.05) in different groups. Red dots represent upregulated genes, blue dots indicate downregulated genes, and black dots denote unchanged genes. These data revealed distinct gene expression profiles of both scaffold groups compared with the intact control, while the differences between the ctrl- and ECM-polyurethane groups were minor. (*n* = 3, biological replicates).

This study aims to further explore the healing mechanisms of ECM-polyurethane scaffolds for repairing large tendon defects. To achieve this, we created a rabbit model with 1 cm SSPT defects, which were then repaired using either ctrl- or ECM-polyurethane scaffolds. One month after the injury, we conducted RNA sequencing (RNA-Seq) and quantitative polymerase chain reaction (qPCR) analyses, comparing the results among the healthy intact control and both scaffolds (Figure 1A). A suture-only repair group was not included in the study, as repairs without scaffold implantation failed at early time points due to inadequate mechanical support [8]. We hypothesized that distinct variations in gene expression profiles during tendon healing would explain the differential healing observed between the ctrl- and ECM-polyurethane scaffold groups.

## 2. Materials and Methods

### 2.1. Fabrication of the Ctrl-Polyurethane and ECM-Polyurethane Scaffolds

The ECM-polyurethane scaffold was fabricated as previously reported [8,15]. In brief, the pre-shaped QHM elastomer was encapsulated within an ECM-containing GelMA hydrogel to form a hybrid core–shell construct. The resulting construct integrated the three primary components: ECM, GelMA hydrogel, and QHM elastomer. To obtain soluble ECM extracts, bovine Achilles tendons were procured from a commercial market in Hong Kong and subjected to urea-based extraction, using the methodology described in our prior work [10]. To prepare the GelMA prepolymer solution, a 10% GelMA (EFL, Building 1, No. 70 East Zhongshan Road, Wuzhong District, Suzhou, Jiangsu, China) solution, with or without ECM (final concentration in the hydrogel: 0.6 mg/mL, as determined by bicinchoninic acid (BCA) assay), was dissolved in phosphate-buffered saline (PBS, Santa Cruz Biotechnolog, Inc., 10410 Finnell Street, Dallas, Texas 75220, USA) containing 0.25% (*w*/*v*) photoinitiator (lithium acylphosphinate salt; EFL). In the ctrl-polyurethane scaffold, the hydrogel layer consisted of GelMA alone, without the addition of ECM, while the hydrogel layer contained the GelMA prepolymer solution functionalized with ECM. A UV-crosslinkable polyurethane elastomer, QHM, was developed based on our previously published protocol [8,11]. To initiate the formation of the ECM-polyurethane scaffold, the surface of the QHM elastomer was first coated with benzophenone, as previously described [8]. Subsequently, the GelMA prepolymer solution (with or without ECM) was gently applied to the freshly treated QHM and exposed to UV irradiation using a UV lamp (EFL; 365 nm, 25 mW/cm^2^) for a duration of 90 s. Crucially, all scaffold crosslinking and assembly steps were performed ex vivo, ensuring the fully fabricated matrix was prepared prior to surgical implantation into the rabbit model.

### 2.2. Establishment of the Large Tendon Defect Model in Rabbits

The rabbit large SSPT defect model (5 mm segmental defect with 10 mm segmental gap) was established based on our previously established protocol [8,15]. All animals were sourced from the Laboratory Animal Services Centre at The Chinese University of Hong Kong. Surgical procedures involving rabbits were performed using sterile techniques, in compliance with the approved protocol (No. 18-003-MIS) of The Chinese University of Hong Kong Animal Experimentation Ethics Committee. A total of eighteen New Zealand White rabbits, both male and female, aged 13 to 16 weeks and with an average body weight of 4 kg, were randomly assigned to the following groups: (1) the ctrl-polyurethane scaffold implantation group (ctrl-polyurethane scaffold), and (2) the ECM-polyurethane scaffold implantation group (ECM-polyurethane scaffold). Each animal underwent an index procedure in which either the left or right SSPT was randomly detached, while the contralateral intact shoulder served as the intact control [8,15].

Prior to the surgical procedure, the rabbits were anesthetized using a mixture of ketamine (35 mg/kg) and xylazine (5 mg/kg) administered via intramuscular injection. The SSPT was accessed through a longitudinal shoulder incision and partial release of the trapezius and deltoid muscles. A rectangular segmental defect measuring 5 mm in length and 10 mm in width was created in the SSPT by full-thickness incision. To mitigate the adverse effects associated with high-tension repairs, either the ECM-polyurethane scaffold or the ctrl-polyurethane scaffold (10 mm width × 10 mm length × 2 mm thickness) was implanted in the rectangular 10 mm gap. The scaffolds were positioned in an inter-positional manner, with each end securely joined to the tendon defect margin utilizing a combination of running–locking and modified Mason–Allen suture techniques [16,17]. The length of the sutured area on the tendon was established as 5 mm. Following implantation, the surgical wound was closed. Postoperative analgesia was maintained with buprenorphine (0.05 mg/kg, subcutaneously) every 8–12 h for 72 h. Free cage activity was allowed after surgery. All animals were housed in the Animal Facility with constant room temperature, humidity, and a 12 h light–dark cycle with ad libitum access to food and water. At a predetermined timepoint, 1 month post-surgery, all six rabbits were euthanized via intraperitoneal injection of pentobarbital at an overdose dosage (>60 mg/kg). The entire SSPT, including the implanted scaffold as well as 3 mm of the surrounding tissue, was subsequently harvested for RNA-Seq, qPCR analysis and histology assessment.

### 2.3. RNA Extraction

At a designated timepoint, 1 month post-surgery, the rabbit SSPT was harvested and immediately placed in 1 mL of RNAIso (Takara, Nojihigashi 7-4-38, Kusatsu, Shiga, Japan 525-0058) before being stored in liquid nitrogen pending RNA extraction and analysis. For RNA extraction, the individual tendon samples were first ground into a fine powder using a mortar and pestle under continuous liquid nitrogen cooling. Subsequently, total RNA was isolated using the Monarch^®^ Total RNA Miniprep Kit (New England Biolabs, 240 County Road, Ipswich, MA 01938-2723, USA) in accordance with the manufacturer’s instructions. RNA purity was assessed using a NanoDrop 2100 (Thermo Fisher Scientific, 168 Third Avenue Waltham, MA 02451, USA), while the RNA integrity was evaluated with the RNA Nano 6000 Assay Kit on the Agilent Bioanalyzer 2100 system (Agilent Technologies, Inc., 5301 Stevens Creek Blvd.Santa Clara, CA 95051, USA). The quality of the RNA samples was determined by measuring the A260/280 nm and A260/230 nm ratios, as well as the RNA integrity number (RIN) values. All RNA samples exhibited high quality, with A260/A280 ratios ranging from 1.8 to 2.0 and RIN values exceeding 5. The isolated RNA samples were stored at −80 °C until the RNA-Seq analysis and qPCR.

### 2.4. RNA-Seq Analysis

RNA-Seq was performed using Illumina’s next-generation sequencing workflow, as previously described [10]. Briefly, at the designated time point, cDNA libraries were prepared by using the NEBNext^®^ Poly(A) mRNA Magnetic Isolation Module and NEBNext^®^ Ultra™ II RNA Library Prep Kit for Illumina (New England Biolabs) according to the manufacturer’s instructions. The sequencing libraries were then validated on the Agilent TapeStation (Agilent Technologies). Samples were sequenced by the Novaseq 2000 system (Illumina, Inc., 5200 Illumina Way, San Diego, CA 92122, USA) using approximately 75 base-pair paired-end RNA-Seq technology with 75–90 million reads per sample.

The sequencing datasets underwent further analysis as follows: (1) Principal component analysis (PCA) was conducted using the plotPCA function from the DESeq2 package. (2) Differentially expressed gene (DEG) analysis was performed using the R package of DESeq2 (v. 1.26.0) [18]. Genes with a cutoff of log_2_ fold change > |1| and false discovery rate (FDR) < 0.05 were considered DEGs. (3) Gene ontology (GO) analysis was carried out using the Protein ANalysis THrough Evolutionary Relationships (PANTHER) classification system [19,20,21] and the Erichr web server [22]. (4) Pathway analysis was performed by using the Kyoto Encyclopedia of Genes and Genomes (KEGG) [23] in the Database for Annotation, Visualization, and Integrated Discovery (DAVID, Resources 6.8, https://davidbioinformatics.nih.gov/, accessed date: 5 December 2025) [24] and the Erichr web server. (5) To investigate the phenotypic switch of stem cells during SPPT healing, single-sample gene set enrichment analysis (ssGSEA) was performed to assess significantly enriched cell phenotypes. This analysis was carried out using the GSVA R package (version 1.50.0) [25]. Gene signatures for stemness markers, tenogenesis, chondrogenesis, osteogenesis, and adipogenesis were selected from the CellMarker database [26] and relevant literature [27,28]. (6) Protein network analyses of the identified proteins were conducted using the Search Tool for Retrieval of Interacting Genes/Proteins (STRING) database (v. 11.5, https://string-db.org/) [29].

### 2.5. qPCR Analysis

Furthermore, to elucidate the changes in gene expression during SSPT healing and to further validate the findings from the RNA-Seq analysis, qPCR was employed to assess the mRNA expression levels of the top expressed DEGs. These markers include those associated with inflammation, tenogenesis, and adipogenesis. Following RNA isolation, cDNA synthesis was conducted using the LunaScript^®^ RT SuperMix Kit (New England Biolabs) in accordance with the manufacturer’s instructions. qPCR was performed utilizing the Luna^®^ Universal qPCR Master Mix (New England Biolabs) on an ABI QuantStudio 7 (Flex) real-time PCR system (Thermo Fisher Scientific, USA). The expression levels of each target gene were normalized to the housekeeping gene *GAPDH*, and relative fold changes were calculated using the ΔΔCT method, with normalization against the intact control at the designated time point. The primer sequences for the target genes are provided in Table 1.

### 2.6. Histological Characterization for Tendon Healing

At designated time points, harvested tendon specimens were immediately fixed in 4% (*w*/*v*) paraformaldehyde (PFA, Sigma-Aldrich, 2909 Laclede Avenue St. Louis, MO 63103, USA) for 48 h at room temperature and, following dehydration, embedded in paraffin blocks and subsequently stained with Hematoxylin and Eosin (H&E), picrosirius red and oil red O in accordance with established standard laboratory protocols, as previously described [30,31]. Digital micrographs of the histochemically stained specimens were acquired utilizing an upright microscope (Nikon Eclipse Ni-U, Nikon Corporation, 1-5-20, Nishioi, Shinagawa-ku, Tokyo 140-8601, Japan). Polarized light microscopy, implemented via the integration of a D-SA Analyzer Slide for simple polarization onto the same optical assembly, was utilized to evaluate specialized fiber architectures [30]. A cohort of at least three animals per experimental condition was utilized for histomorphometric evaluation, with three individual slides evaluated per subject. To ensure robust data representation, four independent regions of interest (ROIs) were isolated on each slide for pixel quantification and subsequent statistical comparisons.

Given the critical role of the collagenous matrix in maintaining structural integrity and facilitating tendon healing [32], picrosirius red staining coupled with circularly polarized light microscopy was utilized to qualitatively and quantitatively characterize collagen fiber content, distribution, and maturation kinetics [32,33,34]. Under polarized light, the characteristic birefringence color hue of collagen fibers shifts based on relative fiber thickness, transitioning from thin, immature green fibers to increasingly thick, mature yellow, orange, and red fibers. Following previously validated protocols [34,35], digital micrographs acquired via polarized light microscopy were processed using ImageJ software (NIH, version 1.54p). The 24-bit RGB images were computationally converted into the 8-bit HSB (hue, saturation, brightness) color space to isolate the hue channel for pixel segmentation. To quantify the relative area of each fiber type, specific color intensity thresholds were applied uniformly across all samples: red fibers were defined by hue values of 0–9 and 230–255, orange fibers by 10–38, yellow fibers by 39–51, and green fibers by 52–128 [32,33,34]. The relative area of each collagen component was subsequently calculated using the following formula:

$$Collagen\;fraction\;cover\;area\left(\%\right)=\frac{Cover\;area\;of\;color\;fraction}{Total\;area}\times 100\%$$

### 2.7. Statistical Analysis

Data are presented as the mean ± standard error of the mean (SEM). All quantitative assays were independently conducted a minimum of three times (*N* in the figure legends represents the number of independent experiments). One-way ANOVA with post hoc Tukey tests was performed using Prism (v9.5.1, GraphPad, 225 Franklin Street. Fl. 26 Boston, MA 02110, USA). *p*-values < 0.05 were considered statistically significant. Statistically significant differences are indicated as follows: *, *p* < 0.05; **, *p* < 0.01; and ***, *p* < 0.001.

## 3. Results

### 3.1. Overview of Transcriptomic Profiles After the Implantation of Ctrl- and ECM-Polyurethane Scaffolds at One Month Post-Surgery

To explore the molecular mechanisms associated with the healing process of the ECM-polyurethane scaffold in the rabbit large tendon defect model, RNA-Seq was performed on the intact control, and the SSPT treated with ctrl- or ECM-polyurethane scaffolds (Figure 1A). A group with suture-mediated repair was not included in this study, as managing a 10 mm full-thickness tear defect in the SSPT through suture repair alone poses significant challenges and was prone to retearing in this animal model [8]. PCA and the heatmap of DEGs demonstrated a clear distinction between the transcriptomes of the different treatment groups, with the greatest variance on PC1 separating the intact control from both treatment groups (Figure 1B,C). DEGs between the different groups are shown in Figure 1D. Between the ctrl-polyurethane scaffold group and the intact control, 4639 DEGs were found, with 2814 upregulated genes and 1825 downregulated genes, as shown in the volcano plot. In addition, 5215 genes were differentially expressed in the ECM-polyurethane scaffold group compared with the intact control, with 3122 upregulated genes and 2093 downregulated genes. Finally, only 69 DEGs were found between the ECM- and ctrl-polyurethane scaffold, with 13 upregulated genes and 56 downregulated genes (Figure 1D). Taken together, these data highlight the distinct gene expression profiles of both scaffold groups compared to the intact control, while the difference in gene expression between the ctrl- and ECM-polyurethane scaffold groups was minor.

**Figure 2:**
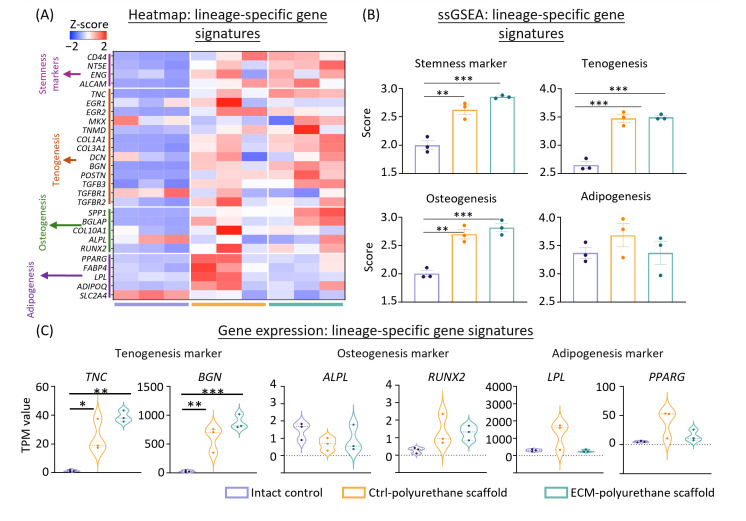
Assessment of cellular phenotypes and lineage markers across the intact control, uncoated polyurethane, and ECM-polyurethane scaffold groups. (**A**,**B**) Heatmap and ssGSEA of lineage-specific gene signatures related to stemness, tenogenesis, osteogenesis, and adipogenesis across different treatment groups. (**C**) Expression levels of selected tenogenesis, osteogenesis, and adipogenesis markers across three groups. The results indicate that both the ctrl- and ECM-polyurethane scaffold groups exhibited increased expression of genes associated with stemness, tenogenesis, and osteogenesis compared to the intact control. (*n* = 3, biological replicates; mean ± SEM; *, *p* < 0.05; **, *p* < 0.01; ***, *p* < 0.001).

Next, the cell phenotypes in each group were characterized using markers associated with stemness, tenogenesis, osteogenesis and adipogenesis. Heatmap analysis of the selected lineage signature genes suggested that both ctrl- and ECM-polyurethane scaffold groups exhibited an increased expression of stemness, tenogenesis, osteogenesis and adipogenesis compared to the intact control. Additionally, the ctrl-polyurethane scaffold group exhibited a reduced expression of stemness and tenogenesis markers and an increased expression of adipogenesis markers compared to the ECM-polyurethane scaffold group (Figure 2A). Moreover, quantitative analysis using ssGSEA showed similar findings. Interestingly, both ctrl- and ECM-polyurethane scaffold groups showed significantly higher ssGSEA scores associated with stemness, tenogenesis and osteogenesis compared to the intact control (Figure 2B). The expression levels of selected tenogenesis marker genes were significantly higher in both the ctrl- and ECM-polyurethane groups compared with the intact control. In contrast, the ctrl-polyurethane scaffold group showed an increasing trend toward adipogenesis marker expression relative to the other two groups. Interestingly, *SCX* (the basic helix–loop–helix transcription factor involved in tendon development) was absent from our gene list, which may be due to incomplete annotation of the rabbit genome in the Ensembl database (Figure 2C). These results indicate that both the ctrl- and ECM-polyurethane scaffold groups show increased expression of genes associated with stemness, tenogenesis, and osteogenesis relative to the intact control, whereas adipogenesis markers exhibited a higher upward trend in the ctrl-polyurethane scaffold groups.

### 3.2. Similar Immune Activation and Extracellular Matrix Remodeling Signatures in Ctrl- and ECM-Polyurethane Scaffold Groups vs. Intact Control

**Figure 3:**
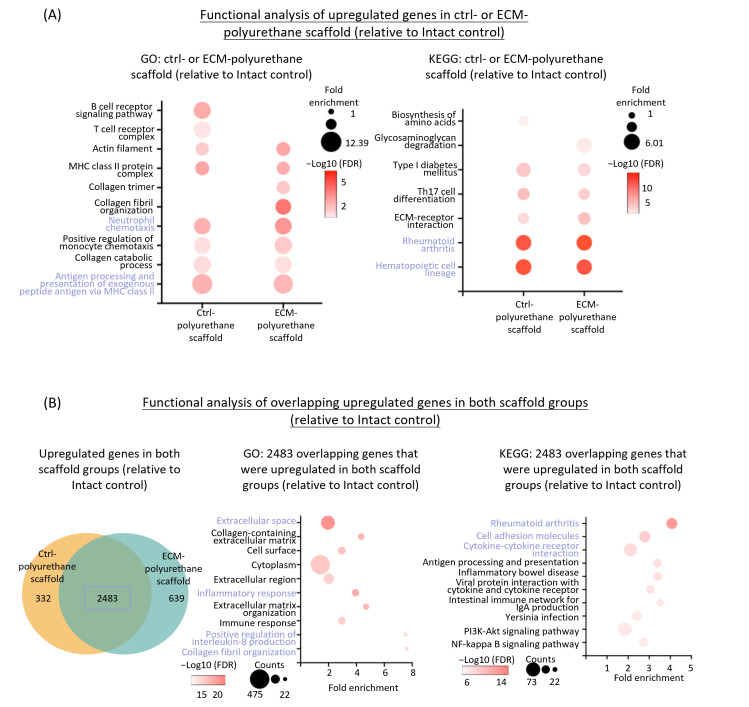
Similarities of transcriptional profiles in ctrl- or ECM-polyurethane scaffold groups relative to the intact control. (**A**) GO enrichment and KEGG pathway analyses of upregulated DEGs in ctrl- or ECM-polyurethane scaffold groups compared to the intact control. The size and color of the bubbles represent the gene counts and FDR value (negative log 10 transformed), respectively, of the DEGs enriched in each term or pathway. Both scaffold groups shared similar GO enrichment patterns and pathways related to immune activation. (**B**) Functional analysis of overlapping upregulated genes in ctrl- and ECM-polyurethane scaffolds, when compared to the intact control. Venn diagram showing 2483 overlapping genes that were upregulated in the ctrl-polyurethane scaffold vs. the intact control and ECM-polyurethane scaffold vs. the intact control. GO enrichment and KEGG pathway analyses of these 2483 overlapping genes. These data demonstrate that ctrl- and ECM-polyurethane scaffolds shared similar GO enrichment patterns and pathways related to inflammatory response and ECM remodeling. (*n* = 3, biological replicates).

The similarities in transcriptomic profiles induced by the two scaffold groups compared to the intact control were explored. GO and KEGG pathway analyses were performed on the DEGs among the three groups (Figure 3). GO analysis revealed that compared to the intact control, the ctrl- and ECM-polyurethane scaffold groups had similar GO enrichment patterns, such as enrichment of genes related to the MHC class II protein complex, and to antigen processing and presentation via MHC class II and neutrophil chemotaxis (Figure 3A). KEGG pathway analysis revealed that compared to the intact control, immune-related pathways such as the pathways associated with hematopoietic cell lineage and rheumatoid arthritis were positively enriched in both ctrl- and ECM-polyurethane scaffold groups (Figure 3A).

Furthermore, we analyzed the DEGs that were commonly upregulated in both the ctrl- and ECM-polyurethane scaffold groups relative to the intact control, identifying a total of 2483 genes (Figure 3B). Enriched GO terms for these 2483 genes are mostly related to extracellular space function (e.g., collagen fibril organization and extracellular space) and inflammatory response (such as positive regulation of interleukin-8 production) (Figure 3B). The KEGG pathway analysis primarily identified pathways associated with inflammatory responses, including rheumatoid arthritis and cytokine–cytokine receptor interactions, as well as ECM-related pathways such as cell adhesion molecules (Figure 3B).

Taken together, these results indicate that both ctrl- and ECM-polyurethane scaffolds elicit broadly similar immune activation GO profiles and inflammatory signaling pathways, relative to intact tendon. This suggests that scaffold-induced healing persists in an ongoing immune activation phase. Additionally, both scaffold groups are enriched in collagen fibril organization-associated processes, indicating that active extracellular matrix remodeling and connective tissue development happen during this period.

### 3.3. ECM-Polyurethane Scaffold Group Induces Collagen-Related Programs, Whereas Ctrl-Polyurethane Group Promotes Lipid Biosynthesis

**Figure 4:**
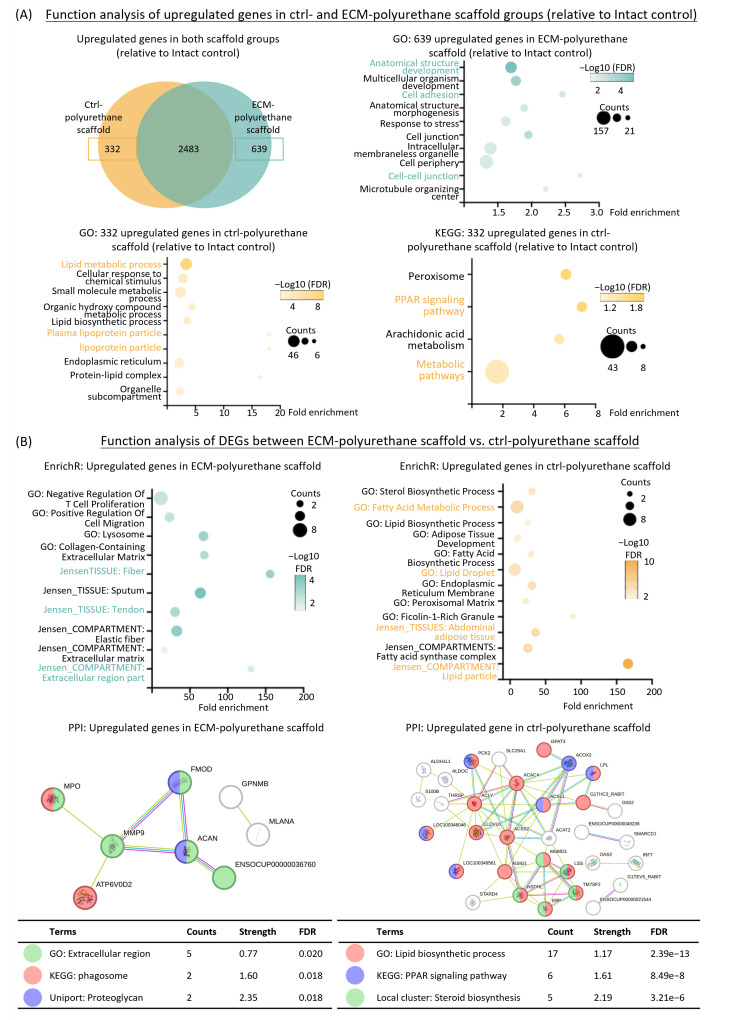
Differences in transcriptional profiles in the ctrl- and ECM-polyurethane scaffold groups. (**A**) Functional analysis of upregulated genes in ECM- and ctrl-polyurethane scaffold groups when compared to the intact control. The Venn diagram shows 332 genes that were only upregulated in the ctrl-polyurethane scaffold group vs. the intact control, while 639 genes were only upregulated in the ECM-polyurethane scaffold group vs. the intact control. GO enrichment analysis of the 639 genes that are only upregulated in the ECM-polyurethane scaffold group showed the enrichment in cell–cell junction and cell adhesion. GO enrichment analysis and KEGG pathway analysis of the 332 genes that are only upregulated in the ctrl-polyurethane scaffold group showed the enrichment in lipid metabolic process and metabolic pathways. The size and color of the bubbles represent the gene counts and FDR value (negative log 10 transformed), respectively, of the DEGs enriched in each term or pathway. (**B**) Functional analysis of DEGs between the ECM-polyurethane scaffold vs. the ctrl-polyurethane group. GO functional analysis and Jensen Tissue database enrichment analysis in ECM- and ctrl-polyurethane scaffold groups. STRING network analysis (confidence score threshold at 0.7 (high)) highlights the interaction networks of the DEGs in ECM-polyurethane scaffold vs. ctrl-polyurethane scaffold groups. Genes are represented as nodes of different colors with unconnected nodes removed. The data demonstrate that the ECM-polyurethane scaffold group was enriched in tendon development-related terms, while the ctrl-polyurethane scaffold group was enriched in adipose tissue development-related terms. (*n* = 3, biological replicates).

Moreover, differential transcriptomic analysis between the ctrl- and ECM-polyurethane scaffold groups was performed. Specifically, gene expression profiling was performed by isolating DEGs uniquely upregulated in the control and ECM-polyurethane scaffold groups, using the intact control as the baseline for comparison (Figure 4A) and by direct comparison of the transcriptomic profiles between the two groups (Figure 4B). It was observed that compared to the intact control, the ECM-polyurethane scaffold group was enriched in GO terms related to cell adhesion, anatomical structure development and cell–cell junction (Figure 4A). Conversely, the ctrl-polyurethane scaffold group showed enrichment for adipogenesis-related terms such as lipid metabolic process, plasma lipoprotein particle, endoplasmic reticulum, as well as the PPAR signaling pathway (Figure 4A).

Direct comparison of DEGs between the two scaffold groups revealed distinct functional enrichments. The ECM-polyurethane scaffold group exhibited significant enrichment for processes related to tendon development (the tendon gene set from the Jensen_TISSUE database) and collagen synthesis such as collagen-containing extracellular matrix. In contrast, the ctrl-polyurethane scaffold group is enriched for terms related to adipose tissue development (the abdominal adipose tissue gene set from the Jensen_TISSUE database) and lipid biosynthesis, including the fatty acid metabolic process and the fatty acid biosynthetic process (Figure 4B). The STRING network analysis also showed similar results. The ECM-polyurethane scaffold group is enriched in the protein cluster related to the extracellular region and proteoglycan, while the ctrl-polyurethane scaffold group is enriched in the protein cluster related to steroid biosynthesis and the lipid biosynthetic process (Figure 4B).

Taken together, these results indicate that ctrl- and ECM-polyurethane scaffolds progress toward different healing processes, tenogenesis and adipogenesis, respectively. Specifically, the ECM-polyurethane scaffold group was enriched in genes related to tendon development and collagen synthesis, while the ctrl-polyurethane scaffold group was enriched in genes associated with adipose tissue development and lipid biosynthesis.

### 3.4. Validation of Immune Activation, ECM Remodeling and Adipogenesis-Related DEGs in Both Scaffold Groups vs. Intact Control via qPCR

**Figure 5:**
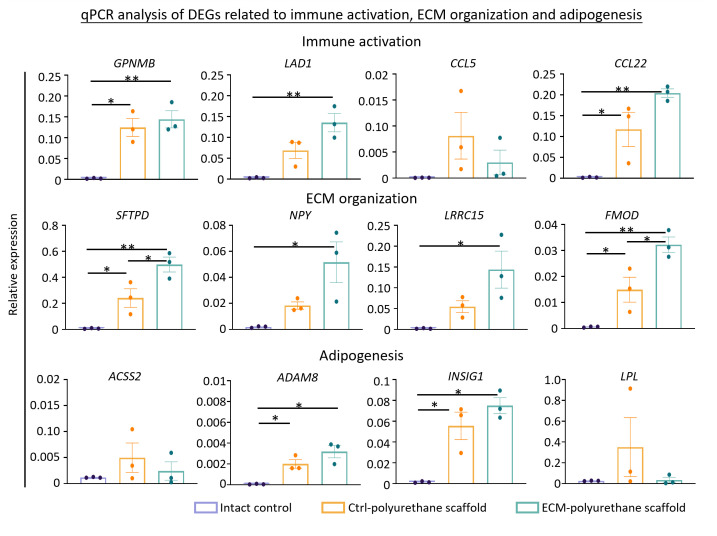
qPCR analysis of selected genes. qPCR analysis of the genes related to the immune activation, ECM remodeling and adipogenesis among the three groups. These data demonstrate that both scaffold groups showed significantly higher expression of immune activation markers such as *GPNMB* and *CCL22*, compared to the intact control. Moreover, the ECM-polyurethane scaffold group is enriched in extracellular reorganization-related genes, such as *SFTPD* and *FMOD*, while the ctrl-polyurethane scaffold group is enriched in lipid biosynthesis-related genes, such as *LPL* and *ACSS2.* (*n* = 3, biological replicates; mean ± SEM; *, *p* < 0.05; **, *p* < 0.01).

To confirm the immune activation, ECM remodeling and adipogenesis within the different scaffold implantation groups, the expression of the top regulated DEGs related to immune response, ECM remodeling and adipogenesis was investigated through qRT-PCR analysis. The expression of the top upregulated genes in ctrl- or ECM-polyurethane scaffold groups vs. the intact control was further analyzed by qPCR to validate the gene expression profiles observed in RNA-Seq analysis (Figure 5). The results showed that, compared to the intact control, the ctrl- and ECM-polyurethane scaffold groups significantly increased the expression of *GPNMB*, a gene encoding for an endogenous glycoprotein, which is related to inflammation and neuroinflammation [36], and *CCL22*, a gene encoding for the CCL22 protein, which is mainly synthesized by M2 macrophages and is also known as the macrophage-derived chemokine (MDC) [37]. In comparison, no significant difference was observed in the expression of these genes between the ctrl- and ECM-polyurethane scaffold groups. Furthermore, genes involved in extracellular reorganization, such as *SFTPD* and *FMOD*, were significantly increased in the ECM-polyurethane scaffold group relative to both the ctrl-polyurethane scaffold group and the intact control. Conversely, genes associated with lipid biosynthesis, such as *LPL* and *ACSS2*, demonstrated a higher expression trend in the ctrl-polyurethane scaffold group. These qPCR results are consistent with the findings from RNA-Seq, where both scaffold groups exhibited significantly higher expression of immune activation markers compared with the intact control. In addition, the ECM-polyurethane scaffold group was enriched for extracellular reorganization-associated genes, whereas the ctrl-polyurethane scaffold group showed an increased trend in lipid biosynthesis-related genes.

### 3.5. Implantation of ECM-Polyurethane Scaffold Enhances Rotator Cuff Tendon Healing Whereas Ctrl-Polyurethane Scaffold Leads to Pronounced Fatty Infiltration

To evaluate the regenerative capacity of ctrl- and ECM-polyurethane scaffolds, histopathological analyses were performed at 1 and 3 months post-surgical repair. Histologically, H&E staining revealed that the architectural quality of the neo-tendon tissue differed substantially between scaffold-treated groups. Semiquantitative cellular orientation analysis demonstrated that cells within the ECM-polyurethane scaffold cohort exhibited significantly higher alignment parallel to the loading axis than those in the ctrl-polyurethane scaffold group at both time points.

**Figure 6:**
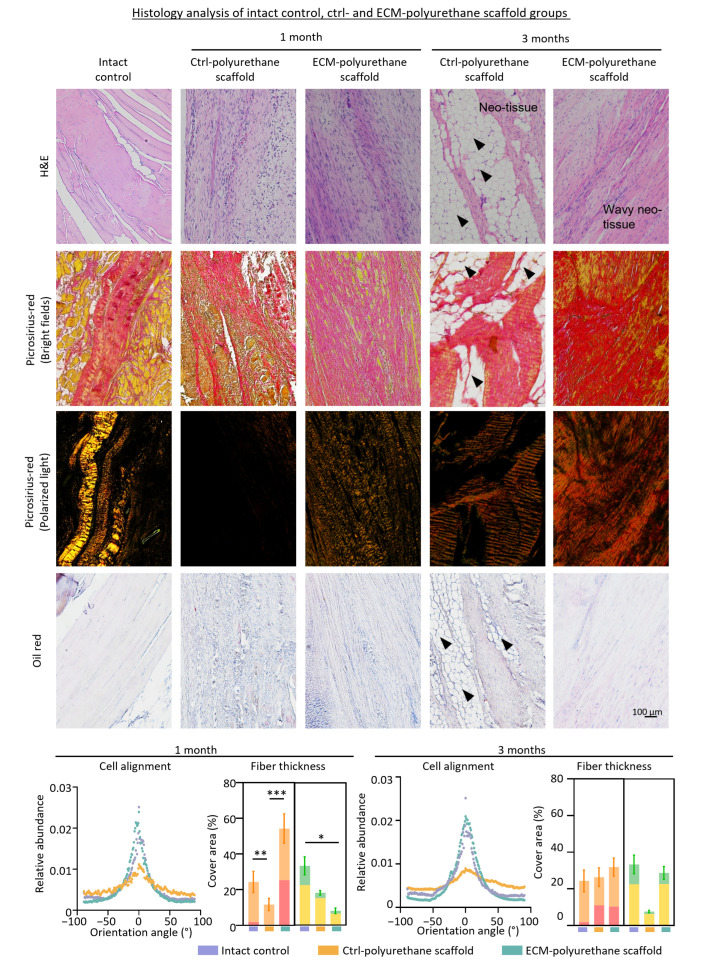
Histological evaluation of ctrl- and ECM-polyurethane scaffolds for large rotator cuff tendon repair. The harvest tendon tissues were evaluated via H&E, picrosirius red and oil red O staining. The ECM-polyurethane scaffold group exhibited superior cellular alignment compared to the ctrl-polyurethane group at both 1 and 3 months post-surgery. Notably, the ctrl-polyurethane group displayed pronounced fatty infiltration at 3 months, as evidenced by extensive lipid vacuoles confirmed through H&E and oil red O staining (black arrow: lipoid infiltration). Additionally, polarized light microscopic images of picrosirius red-stained sections revealed that the ECM-polyurethane group maintained a wavy, highly organized extracellular matrix structure with enhanced birefringence, characterized by an increased density of thicker collagen fibers at 1 month compared to the control group (Thick fiber: orange-to-red, Thin fiber: green -to-yellow). (*n* = 3, biological replicates; mean ± SEM; *, *p* < 0.05; **, *p* < 0.01; ***, *p* < 0.001.).

Notably, the ctrl-polyurethane group exhibited pronounced fatty infiltration (FI), as evidenced by extensive lipid vacuoles confirmed via H&E and oil red O staining at 3 months. Conversely, the ECM-polyurethane group maintained a highly organized, wavy, and elongated tissue morphology. Polarized light microscopic images of picrosirius red-stained sections were utilized to evaluate collagen fiber diameter and organization based on its birefringent nature [38]. While the ctrl-polyurethane group displayed diminished birefringence characteristic of disordered scar tissue, retaining a predominance of thin, immature yellow fibers, the ECM-polyurethane cohort exhibited a pronounced accumulation of mature, thick orange-to-red fibers (Figure 6). Collectively, these histomorphometric data demonstrate that ECM-polyurethane scaffolds significantly improve cellular and extracellular matrix organization while enhancing mature collagen deposition compared to unsupplemented ctrl-polyurethane scaffolds, which exhibited pronounced fatty infiltration.

## 4. Discussion

In our prior work, we developed an ECM-polyurethane scaffold with a core–shell structure integrating an ECM-rich hydrogel with a mechanically robust QHM polymer. This scaffold promoted robust tendon regeneration and facilitated significant functional recovery in large tendon defects. In the present study, we further elucidated the healing mechanisms of the ECM-polyurethane scaffold through transcriptomic analysis. The findings revealed two key patterns: (1) both ctrl- and ECM-polyurethane scaffold groups displayed comparable GO enrichment patterns and pathways associated with the inflammatory response when compared to healthy intact tendon; (2) pairwise comparison between the ctrl- and ECM-polyurethane scaffold groups indicated that the ECM-polyurethane scaffold group was enriched in genes related to tendon development and extracellular matrix composition, whereas the ctrl-polyurethane scaffold group was enriched in genes associated with adipose tissue development.

Biomaterial scaffolds serve as a critical component in rotator cuff repair, providing enhanced mechanical strength, reducing healing stress, and promoting cell proliferation and migration [39]. Our innovative ECM-polyurethane scaffold leverages the synergy between biomechanical (QHM polymer core) and biological (ECM) augmentation, and presents a promising strategy to improve tendon repair outcomes [8]. Specifically, the QHM polymer core exhibits biomechanical properties comparable to those of the human SSPT. It demonstrates excellent suture retention, robust tensile strength, gradual degradation, low cytotoxicity, and favorable biocompatibility [8,11]. Additionally, its degradation products do not disrupt cellular proliferation or differentiation. Long-term in vivo implantation confirms excellent histocompatibility without adverse pathology, demonstrating that the QHM scaffold delivers stable mechanical support alongside a biocompatibility profile. Our previous study demonstrated that the urea-extracted tendon ECM contains multiple key ECM proteins, including decorin, biglycan, and fibromodulin, which are essential for collagen formation and tendon differentiation [10]. Furthermore, ECM-treated human adipose-derived stem cells (hASCs) expressed a range of genes that are associated with ECM and cell activity processes, which may partially explain the observed enhancements in cell proliferation and tenogenic differentiation of these cells [10,40]. Moreover, in a rat patellar tendon injury model, the ECM-loaded scaffold demonstrated the ability to promote functional tendon regeneration [30]. Finally, our previous in vivo data underscore the critical role of ECM in the ECM-polyurethane scaffold design for rotator cuff tendon repair. The implantation of the ECM-polyurethane scaffold, unlike the ctrl-polyurethane scaffold, facilitated early and rapid alignment of tendon fibers, which was strongly correlated with increased tendon stiffness at the 3-month time point [8].

Tendon healing is a complex, protracted process that occurs in three overlapping stages. The inflammation stage is initiated immediately after injury. This stage is marked by hematoma formation and infiltration of inflammatory cells at the injury site [41]. Approximately 1–2 weeks post-injury, the cell proliferation stage predominates. This stage is characterized by heightened synthetic activity by macrophages and tenocytes and a peak in collagen type III synthesis [41,42]. Water content and glycosaminoglycan levels remain elevated. The final remodeling phase commences 1–2 months after injury and lasts over a year. This phase features predominant collagen type I synthesis and increased ECM alignment [41]. In this study, our transcriptomic analysis revealed distinct gene expression profiles for the ctrl- and ECM-polyurethane scaffold groups compared to the intact control (Figure 1B–D). This may indicate that, compared to the healthy tendon, both scaffold-implanted tendons are still in the active healing process at week 4 post-surgery time-point. Moreover, both scaffold groups displayed similar GO enrichment patterns and pathways related to the inflammatory response, including the positive regulation of interleukin-8 production and pathways related to rheumatoid arthritis, and cytokine–cytokine receptor interactions. These findings suggest that both scaffold implantation groups are characterized by an activated immune response, the initial healing stage after injury (Figure 3). Additionally, both groups were enriched in ECM remodeling-related GO terms and pathways, such as collagen fibril organization, suggesting progression toward the second stage of healing (Figure 3B).

Tendons typically exhibit a reparative rather than regenerative response after injury, which is characterized by scar formation and a disorganized ECM with inferior tensile strength. Additionally, FI following tendon injury is a common occurrence that adversely affects the outcomes of the healing process [6]. Fang et al. performed RNA sequencing on RCT tissue samples obtained from a bilateral supraspinatus tear-repair rat model. Their KEGG enrichment analysis revealed that the most significant alteration in RNA expression was associated with non-alcoholic fatty liver disease. Additionally, their histological examination of the RCT tissue identified FI at 1, 2, and 4 weeks post-surgery [6]. Moreover, Vasquez-Bolanos et al. [43] investigated the temporal transcriptional changes during rotator cuff tendon repair. They found that rotator cuff repair does not elicit the anticipated growth or regenerative response. Instead, the repair is primarily associated with metabolic and energetic changes at week 1 post-repair, increased adipogenesis at week 4, and a dysregulated stress response at week 8. Similar results were observed in our study. Although gene expression differences between the ctrl- and ECM-polyurethane scaffold groups were relatively minor (69 DEGs identified between the two groups), the result showed that ctrl- and ECM-polyurethane scaffolds progress toward different healing processes, tenogenesis and adipogenesis, respectively. The ctrl-polyurethane scaffold group nonetheless showed enrichment of genes associated with adipose tissue development, including fatty acid and lipid metabolic processes and the PPAR signaling pathway. In contrast, the ECM-polyurethane scaffold group showed enrichment in genes associated with tendon development, such as the tendon gene set from the Jensen_TISSUE database and the collagen-containing extracellular matrix GO terms (Figure 4). Therefore, in conjunction with our previous findings [8], the ECM-polyurethane scaffold group showed superior tendon-healing capacity and began to exhibit signs of tendon regeneration at week 4 post-surgery, compared with the control-polyurethane group (Figure 4 and Figure 5). These divergent transcriptomic profiles suggest that the control polyurethane scaffold lacks the appropriate biochemical signaling, resulting in an upregulation of lipid metabolic pathways and a shift toward a fibro-adipogenic lineage. Conversely, the ECM-polyurethane scaffold shows upregulation of structural extracellular matrix networks and tenogenic-associated gene expression, suggesting that the ECM components provide inductive cues that promote tenogenesis and proper tissue formation. Furthermore, this molecular divergence was structurally mirrored in our histological evaluations (Figure 6), where the ECM-polyurethane scaffold successfully drives a well-aligned, mature collagen matrix, effectively suppressing the degenerative adipogenic phenotype that plagued the unsupplemented control scaffold matrix.

Our transcriptomic and histological findings establish a novel paradigm wherein the ECM-polyurethane framework effectively channels early-stage post-operative signaling away from degeneration and toward functional tissue synthesis. This robust, translationally scalable platform represents a significant leap forward in musculoskeletal biomaterials engineering, successfully transforming a traditionally compromised healing trajectory into a highly predictable pathway for complete, functional rotator cuff regeneration.

This study has several important limitations. First, the sample size may limit the overall statistical power of the analyses; however, the deployment of separate cohorts (*n* = 6 for RNA-Seq and *n* = 12 for histology) was a deliberate design choice to prevent technical interference between the assays. This strategy was strictly guided by feasibility, animal welfare considerations, and ethical constraints inherent to in vivo rabbit models. Moreover, we assessed only the 4-week time point in RNA-Seq and qPCR analysis because it represents a clinically relevant stage of early post-surgical tendon healing, during which scaffold-mediated effects on matrix remodeling are expected to occur. Our previous work and our histology data confirmed that the ECM-polyurethane scaffold group formed tendon-like ECM structures with a wavy configuration, in contrast to the disorganized scar tissue observed in the ctrl-polyurethane group at 3 months post-surgery. We will perform long-term post-surgical evaluations (such as 6 to 12 months) in future large preclinical animal studies. Additionally, this study used paraffin-embedded sections rather than frozen blocks, as standard processing solvents extract lipids and compromise oil red O staining. Nonetheless, the presence of extensive, well-defined empty vacuoles within the mid-substance of the control tendons provides clear morphological evidence of localized tissue degeneration [44,45,46,47].

## 5. Conclusions

Our findings suggest that the polyurethane scaffold, while exhibiting biomechanical features similar to human tendons, can elicit different healing responses depending on the presence or absence of tendon-derived ECM. This highlights the significance of incorporating both robust regenerative signals and stringent biomechanical support in bioscaffold design, which is essential for addressing substantial defects in tendons and other load-bearing tissues.

## Figures and Tables

**Table 1:** Sequences of primers used in qPCR.

	Forward Primers (5′ to 3′)	Reverse Primers (5′ to 3′)
*GAPDH*	GCAAAGTGGATGTTGTCGCC	TGATGACCAGCTTCCCGTTC
*GPNMB*	TACAAGCTGGGACGGTGTTC	ACACGGTCACCTCCATAAGC
*LAD1*	CCTTCTCTAACTTTTCCGCTCG	TCCCTGCCTGGAAATCGAC
*CCL5*	CTATACCAGCGGCAAGTGCT	TCTTCTCTGGGTTGGCACAC
*CCL22*	GGCCTTTAGTGCTCCTCCAG	TTCTCCCGTTCGTCCACTTG
*SFTPD*	CTCTAGGTCGTTGTGCAACTCA	AAACAACCCCCTCAGTGTCTG
*NPY*	CCACAATGCTCGGGAACAAG	CAGGCATACAAGCAGGGACA
*LRRC15*	CGACTCTACGACAACCCCTG	TTGTTGAGCAGGAGCCAGTT
*FMOD*	AGGCTCCTGGTTAGGAATTTGG	AAGTGAGCTCTGCTGAGAGTG
*ACSS2*	TGTCCAGGGTAAGCTGAAAGAG	TGCCACCACAAGTCAATCCC
*ADAM8*	CATCTCACGAACTCTGGTGGTT	AGAAGCCACAGTCTGGGAAGT
*INSIG1*	AGGGGGCAGTTTCCTGAGTA	AGCACCCTCAACCTATCCCT
*LPL*	GTGTAACATCGGGGAAGCCA	TTCGTGGGAGCACTTCACAA

## Data Availability

The datasets used and/or analyzed during the current study are available from the corresponding author on reasonable request.
